# Prevalence and Fate of Carbapenemase Genes in a Wastewater Treatment Plant in Northern China

**DOI:** 10.1371/journal.pone.0156383

**Published:** 2016-05-26

**Authors:** Fengxia Yang, Daqing Mao, Hao Zhou, Yi Luo

**Affiliations:** 1 School of Environmental Science and Engineering, Tianjin University, Tianjin, China; 2 College of Environmental Science and Engineering, Ministry of Education Key Laboratory of Pollution Processes and Environmental Criteria, Nankai University, Tianjin, China; Amphia Ziekenhuis, NETHERLANDS

## Abstract

Carbapenemase-producing strains of bacteria, which were primarily found in the medical field, have increasingly been found in the environment, thus posing potential risks to public health. One possible way for carbapenemase genes to enter the environment is via wastewater. Therefore, the goal of this study was to determine the occurrence and fate of five high-risk carbapenemase genes in a wastewater treatment plant (WWTP) in northern China using real-time qPCR. Results showed that the *bla*_KPC-2_, *bla*_GES-1_, and *bla*_IMP-1_ genes prevailed throughout all processing stages (even in the chlorination disinfection unit) in the WWTP, whereas the *bla*_VIM-2_ and *bla*_OXA-48_ genes were not detected in all samples. Worryingly, considerable amounts of carbapenemase genes ((1.54 ± 0.61) × 10^3^ copies/mL to (2.14± 0.41) × 10^5^ copies/mL) were detected in WWTP effluent samples, while the majority of the carbapenemase genes were transported to the dewatered sludge with concentrations from (6.51 ± 0.14) × 10^9^ copies/g to (6.18 ± 0.63) × 10^10^ copies/g dry weight. Furthermore, a total of 97 KPC-2-producing strains, belonging to 8 bacterial genera, were isolated from the WWTP. Sequencing of 16S rRNA revealed that most of KPC-2 producing isolates were opportunistic pathogens, including *Klebsiella spp*. (10.3%), *Enterococcus spp*. (11.3%), *Acinetobacter spp*. (19.6%), *Escherichia spp*. (12.4%), *Shigella spp*. (17.5%), *Stenotrophomonas spp*. (10.3%) and *Wautersiella spp*. (9.3%). Moreover, *bla*_KPC-2_ genes were identified for the first time in *Paenibacillus spp*. isolates (an indigenous bacteria), indicating an increased risk of horizontal transfer between clinical pathogens and environmental bacteria. Indeed, a conjugation experiment demonstrated transfer of the *bla*_KPC-2_ gene to an *E*.*coli* J53 strain from a *Klebsiella* strain isolated from the WWTP. To our knowledge, this is the first study to obtain *Paenibacillus spp*. isolates carrying the carbapenemase gene and to quantify the abundance of carbapenemase genes in the environment.

## Introduction

Carbapenems are usually used as the antibiotics of last resort for most serious bacterial infections [1–4]. However, carbapenem resistance in clinical isolates has emerged with the increasing use of carbapenem antibiotics [5]. Previous studies indicated that the most common mechanism of carbapenem resistance involved the expression of carbapenemase [6]. Carbapenemase-producing bacteria, especially those producing New Delhi metallo-β-lactamase-1 (NDM-1) and Klebsiella pneumoniae carbapenemase (KPC), are capable of resisting a wide range of β-lactams, including almost all carbapenem antibiotics [7], which poses serious threats to human health [8].

The emergence of carbapenemase-producing isolates, as a therapeutic challenge, has been increasingly reported worldwide [9, 10]. Among the carbapenemase genes, the most prevalent are the *bla*_KPC-2_, *bla*_GES-1_, *bla*_IMP-1,_ *bla*_VIM-2_ and *bla*_OXA-48_ genes, which are propagated and disseminated worldwide [9, 11–14]. The carbapenemase KPC-2 (Ambler class A) was first detected in 1996 from a clinical isolate of *Klebsiella pneumoniae* [15], and GES-1, another class A carbapenemase, was first found in 1998 from a *Klebsiella pneumomiae* isolate [16]. Likewise, Ambler class D carbapenemases (especially OXA-48) are frequently detected in *Acinetobacter spp*. and *Enterobacteriaceae spp*. [14,17]. Moreover, carbapenemase IMP-1 and VIM-2 (Ambler class B) are the most common metallo-β-lactamases (MBLs) among clinical isolates [18,19]. More worryingly, carbapenemase genes located on plasmids could disseminate through horizontal gene transfer among different species of bacteria [6,9], contributing to the global spread of carbapenem-resistant bacteria.

By now, carbapenem resistance has become a worldwide concern, and studies on the detection of carbapenemase-producing isolates in clinical settings are increasingly being reported [20–22]. Carbapenemase genes have become endemic in many countries, including Portugal [11], Austria [23], Switzerland [24], Turkey [25] and Brazil [26]. Notably, some carbapenemase-producing isolates have been recovered from the non-clinical environment, including coastal recreational water [26], activated sewage sludge [23], rivers [11] and lakes [24]. However, these investigations were mostly limited to a culture-based approach that can only characterize less than 5% of the bacterial spectrum [27]. Recent studies on the incidence and dissemination of β-lactams in wastewater treatment plant (WWTP) have relied on other methods, such as enumeration of resistant bacteria, antimicrobial susceptibility testing, nucleotide sequence analysis, and functional metagenomic analysis [28–31]. However, there is a lack of data on the abundance of some carbapenemase gene (i.e., *bla*_KPC-2_, *bla*_GES-1_, *bla*_IMP-1_, *bla*_VIM-2_ and *bla*_OXA-48_) in the non-clinical environment. Determining this abundance is critical to understanding the fate and spread-pathway of carbapenemase genes in the environment.

The purpose of this study was to investigate the prevalence, fate and removal of carbapenemase genes (*bla*_KPC-2_, *bla*_GES-1_, *bla*_IMP-1_, *bla*_VIM-2_ and *bla*_OXA-48_) and carbapenemase-producing isolates in WWTP and the dissemination of resistance among environmental bacteria. To our knowledge, this is the first study on the prevalence and fate of these carbapenemase genes in various stages of waste water treatment.

## Materials and Methods

### Sample collection

**Fig 1:**
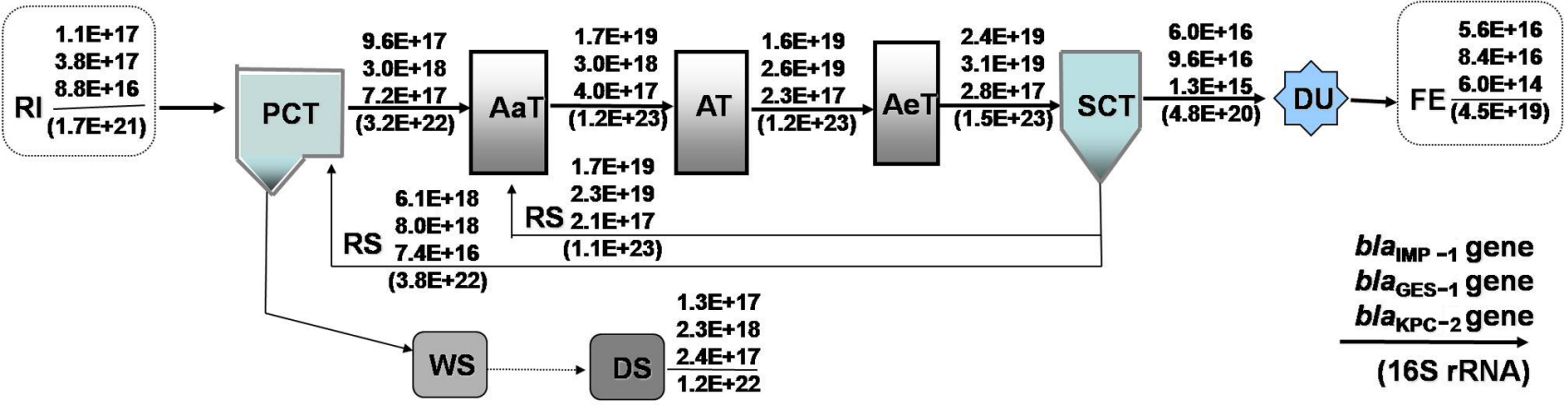
Process configuration and gene flow (copies per day) through a wastewater treatment plant (WWTP) in northern China. The daily abundance of carbapenemase genes is shown above the arrow, and abundance of 16S rRNA genes (in parentheses) are given below the arrow. Abbreviations: RI, raw influent; PCT, primary clarifier tank; AaT, anaerobic tank; AT, anoxic tank; AeT, aerated tank; SCT, second clarifier tank; DU, disinfection unit; FE, final effluent; WS, waste sludge; DS, dewatered sludge.

No specific permissions were required for the proposed activities in the study locations, and we confirm that the field studies did not involve endangered or protected species. Both wastewater and sludge samples were collected from a WWTP in Tianjin, China. This plant serves a large urban catchment of approximately one million people. The WWTP has a primary treatment step with conventional activated sludge, including the main steps of pre-sedimentation and anaerobic-, anoxic-, and aerated-treatments, followed by a second clarifying treatment. The main steps in the WWTP process are presented in Fig 1. Wastewater entering into the treatment plant is a mixture of domestic, industrial wastewater. All samples were collected in triplicate from the effluent of various units every 2 h for a 24 h period to avoid confounding effects associated with hydraulic loading fluctuations, stored in sterile containers and transported to the laboratory on ice for immediate processing and further experimentation.

### DNA extraction and PCR and qPCR of carbapenemase genes

Wastewater samples were vacuum-filtered with 0.22 μm filters, and DNA extraction was performed using an ultraclean water DNA kit (MoBio Laboratories Inc.) following the manufacturer’s instructions. Sludge samples were subdivided into two equivalent subsamples. One subsample was lyophilized to calculate the percentage of moisture for further DNA quantification. DNA was extracted from another subsample using a soil DNA isolation Kit (MoBio Laboratories Inc.). To minimize PCR inhibition, the extracted DNA of each sample was further purified using a DNA pure spin kit (Vigorousbio, Beijing, China). Meanwhile, *E*. *coli* DH5α, cloned with the CESA9 gene as an internal standard, was used to determined DNA extraction efficiency, as previously described [32]. All DNA samples were stored at -20°C until further analysis.

The presence of five carbapenem-resistance genes (*bla*_KPC-2_, bla_GES-1_, *bla*_IMP-1_, *bla*_VIM-2_ and *bla*_OXA-48_) was investigated by PCR using a Biometra thermocycler (Biometra T Gradient, Germany), followed by DNA cloning and sequencing using a pEASY-T3 Cloning Kit (Transgene Beijing, China). DNA extracts were diluted from 10 to 50-fold. PCR with 16S rRNA was used to optimize the template concentration. For each DNA extract, duplicate PCR tubes were analyzed to detect the target genes. The target genes that were amplified using the primers designed herein had 100% identity to the reported sequences available in the Genbank database (geneID: 15564405 for *bla*_KPC-2_; geneID: 9481713 for *bla*_GES-1_) by sequence alignment (with Genbank database), suggesting the validity of the PCR detection. Quantification of the bacterial 16S rRNA, *bla*_KPC-2_, *bla*_GES-1_, *bla*_IMP-1_, *bla*_VIM-2_ and *bla*_OXA-48_ genes was carried out by qPCR amplifications, performed on a Bio-Rad iQ5 instrument (Bio-Rad Company, CA, USA). Calibration standard curves for positive controls were generated as previously described [32]. Negative controls, containing all of the components of the PCR mixture without DNA template, were used in every PCR run. The copy numbers of the target genes were determined in triplicate for each sample.

All qPCR assays were implemented in 96-well plates with a final volume of 25 μL of reaction mixture (TransStart Top Green qPCR SuperMix, TransGen), including 1 μL of template DNA and 0.25 μM of each primer. The qPCR reaction proceeded as follows: initial denaturing at 95°C for 5 min, followed by 40 cycles of 15 s at 95°C, 1 min at the annealing temperature, 30 s at 72°C, and a final melt curve stage with temperatures ramping from 55°C to 95°C (0.5°C per read, 30 s hold) to corroborate the specificity of qPCR products. High R^2^ values (0.997 ~ 0.999) and high efficiencies (80% ~ 105%) were obtained over at least 6 orders of magnitude in all qPCR assays, indicating the validity of these quantifications. Details of the PCR and qPCR reactions can be found in our previous publications [32]. The primers [33–35] used in this study are listed in Table 1.

**Table 1:** PCR primers used in this study.

Target Gene	Primer	Sequence (5’-3’)	Traditional PCR annealing temp. (°C)	qPCR annealing temp. (°C)	Ampliconsize (bp)	Source
***bla***_**KPC-2**_	K-FW	ATGTCACTGTATCGCCGTCT	55	-	893	[33]
	K-RV	TTTTCAGAGCCTTACTGCCC				
**S-*bla***_**KPC-2**_	SK-FW	GCTTCCCACTGTGCAGCTCATTC	66.1	66.1	213	This study
	SK-RV	CGCCCAACTCCTTCAGCAACAAATTG				
**S-*bla***_**GES-1**_	SG-FW	ATGGCACGTACTGTGGCTAA	56	56	287	This study
	SG-RV	TGACCGACAGAGGCAACTAAT				
***bla***_**IMP-1**_	I-FW	GGAATAGAGTGGCTTAAYTCTC	50	50	232	[34]
	I-RV	GGTTTAAYAAAACAACCACC				
***bla***_**VIM-2**_	V-FW	GTTTGGTCGCATATCGCAAC	60	-	382	[34]
	V-RV	AATGCGCAGCACCAGGATAG				
***bla***_**OXA-48**_	O-FW	GCGTGGTTAAGGATGAACAC	47	47	438	[34]
	O-RV	CATCAAGTTCAACCCAACCG				
**16S rRNA**	16S-FW	CGGTGAATACGTTCYCGG	58	57.5	126	[35]
	16S-RV	GGWTACCTTGTTACGACTT				
**16S rRNA**	27F	AGAGTTTGATCCTGGCTCAG	56	-	1466	[36]
	1492R	GGTTACCTTGTTACGACTT				

### Isolation of carbapenemase-producing bacteria

Dual resistance Luria-Bertani (LB) agar plates were used to isolate carbapenem-resistant strains from the WWTP samples. Ampicillin and imipenem were used as indicator antibiotics, and the screening breakpoints were set at 100 mg/L and 1 mg/L, respectively. For screening, the complete samples were plated on the screening LB agar plates, and incubated for 24 h at 37°C. The number of colony forming units (CFUs) were counted on plates that contained between 30 and 100 colonies. All samples were made in triplicate. In order to compare bacterial changes in various treatment units, the numbers of isolates at every stage were represented as the CFUs per 100 μL samples for each sampling site. To avoid selection of only carbapenem-resistant bacteria, the samples were also cultured without antibiotics.

Each isolate was subcultured in fresh LB broth at 37°C for 24 h, and DNA was extracted using the E.Z.N.A. Bacterial DNA Kit (OMEGA, USA), following the manufacturer's instructions. Extracted DNA was further purified using a DNA Pure-Spin Kit (Vigorousbio, Beijing, China) to minimize PCR inhibition. The presence of five relevant carbapenem-resistance genes was investigated by PCR. All isolates were identified by 16S rRNA sequencing using specific primers [36]. The susceptibility of the isolated bacteria to antibiotics was determined by the broth microdilution method as described by the Clinical and Laboratory Standards Institute [37]. The minimum inhibitory concentrations (MICs) for nine antibiotics (imipenem, meropenem, ceftazidime, ampicillin, tetracycline, streptomycin, gentamicin and ciprofloxacin) were tested using the broth microdilution method with 14 gradient dilutions followed by multiple proportional dilutions (256, 128, 64, 32, 16, 8, 4, 2, 1, 0.5, 0.25, 0.12, 0.06, 0.03 mg/L), as recommended by the Clinical and Laboratory Standards Institute [37]. The antibiotic concentration resulting in complete (100%) inhibition of bacterial growth, as compared with the control group, was the MIC value of the isolate to that antibiotic.

### Bacterial conjugation and plasmid characterization

In order to study the transferability of the bla_KPC-2_ gene, mating-out assays were performed using an azide-resistant *E*.*coli* J53 strain (kindly provided by Professor Minggui Wang) as the recipient and one *bla*_KPC-2_-positive *Klebsiella* isolate from the sludge sample as the donor, as described previously [38]. Briefly, the recipient and donor were incubated separately in LB broth at 37°C; overnight cultures were adjusted to 10^8^ cells/ml with fresh LB broth. The donor and the recipient were then mixed at a donor-to-recipient volume ratio of 1:1. The transconjugants were selected on LB agar plates containing sodium azide (100 mg/L) and imipenem (1 mg/L). To verify plasmid transfer, plasmid DNA was extracted from the transconjugants and environmental isolates using a plasmid extraction kit (OMEGA, USA), according to the manufacturer’s instructions. PCR and DNA sequencing were conducted to confirm whether the *bla*_KPC-2_ gene had been transferred. Plasmid incompatibility groups were determined using the PCR-based replicon typing method, as described previously [39]. The transfer frequency was expressed as the numbers of transconjugants per recipient cell.

## Results and Discussion

### Occurrence and fate of carbapenemase genes in various processing stages of a WWTP

The *bla*_KPC-2_, *bla*_GES-1_ and *bla*_IMP-1_ genes were found at each processing stage of the WWTP, including raw influent (RI), primary clarifier tank (PCT), anaerobic tank (AaT), anoxic tank (AT), aerated tank (AeT), second clarifier tank (SCT) and final effluent (FE), which indicated the prevalence of these carbapenemase genes in the WWTP. VIM-2 and OXA-48 subtypes were not detected in any samples. The absolute abundances of carbapenemase genes at different processing stages are shown in Table 2. Notably, the *bla*_KPC-2_, *bla*_GES-1_ and *bla*_IMP-1_ genes were detected at considerable concentrations ((1.54 ± 0.61) × 10^3^ copies/mL, (2.14 ± 0.41) × 10^5^ copies/mL, (1.44 ± 0.43) × 10^5^ copies/mL) in the FE of the WWTP, which represent 0.7%, 22.8% and 51.8% of influent gene levels, respectively. Furthermore, the concentrations of the *bla*_KPC-2_, *bla*_GES-1_ and *bla*_IMP-1_ genes in the dewatered sludge (DS) was as high as (6.51 ± 0.14) × 10^9^ copies/g, (6.18 ± 0.63) × 10^10^ copies/g, and (3.49 ± 0.81) × 10^9^ copies/g, respectively, which raises the possibility of propagation to indigenous bacteria in the soil during fertilization or landfill operations. Considering the different nature of the samples, carbapenemase gene levels were also normalized to 16S rRNA gene levels to place abundance data into context. The relative concentrations of carbapenemase genes in DS were not significantly more enriched than in water samples, which implied that fluctuations of carbapenemase gene concentrations in different samples primarily resulted from changes in total microbial abundance.

**Table 2:** The abundance of carbapenemase and 16S rRNA genes and DNA extraction recoveries for each samples from the WWTP.

WWTP	Gene (copies/ml water or g sludge dw)	Stages in WWTP							
		RI	PCT	AaT	AT	AeT	SCT	FE	DS
	***bla***_**KPC-2**_	(2.2±0.8)E+05	(1.8±0.3)E+06	(1.0±0.2)E+06	(5.8±1.0)E+05	(7.1±1.3)E+05	(3.2±1.0)E+03	(1.5±0.6)E+03	(6.5±0.1)E+09
	***bla***_**GES-1**_	(9.5±0.4)E+05	(7.5±0.7)E+06	(7.5±0.8)E+06	(6.5±0.7)E+07	(7.7±0.8)E+07	(2.4±0.6)E+05	(2.1±0.4)E+05	(6.2±0.6)E+10
**genes**	***bla***_**IMP-1**_	(2.8±0.9)E+05	(2.4±1.0)E+06	(4.3±0.3)E+07	(4.0±0.1)E+07	(5.9±0.2)E+07	(1.5±0.4)E+05	(1.4±0.4)E+05	(3.5±0.8)E+09
	***bla***_**VIM-2**_	ND.	ND.	ND.	ND.	ND.	ND.	ND.	ND.
	***bla***_**OXA-48**_	ND.	ND.	ND.	ND.	ND.	ND.	ND.	ND.
	**16S rRNA**	(4.3±1.2)E+09	(8.0±1.1)E+10	(3.1±0.9)E+11	(3.1±0.8)E+11	(3.7±0.7)E+11	(1.2±0.8)E+09	(1.1±0.2)E+08	(3.2±0.8)E+14
**DNA extraction recoveries**		0.58	0.53	0.55	0.69	0.50	0.59	0.74	0.48

Abbreviations: RI: raw influent, PCT: primary clarifier tank, AaT: anaerobic tank, AT: anoxic tank, AeT: aerated tank, SCT: second clarifier tank; FE: final effluent; and DS: dewatered sludge; dw: dry weight. The amplification of standard plasmids carrying the target gene to establish the standard curve was used as a positive control for the detection of target genes in the samples; negative controls were below the limit of detection (8–20 copies per 25 μl of reaction mixture) in all assays.

Variations in carbapenemase genes (*bla*_KPC-2_, *bla*_GES-1_ and *bla*_IMP-1_ gene) at different processing stages in the WWTP are shown in Table 2; the presence of these genes followed a similar trend to that of 16S rRNA genes. A positive correlation (*p* < 0.01) was found between the abundance of carbapenemase genes and 16S rRNA genes in the WWTP, demonstrating that the fluctuation of carbapenemase gene concentrations through various processing stages may be attributable to microbial growth. There was significant replication (*p* < 0.05) during the biological treatment stages for these carbapenemase genes, primarily because the recycled and activated sludge increased bacterial density and microbial biomass. These findings are in accord with a previous study [40,41].

From Table 2, it can also be seen that there was a relatively high reduction in carbapenemase genes and 16S rRNA genes in SCT, where the removal efficiency of these genes was higher than 99%. There were approximately 2 orders of magnitude reductions in carbapenemase genes in SCT compared with AeT. High concentrations of carbapenemase genes were settled and transferred into sludge, which increases the risk of propagating carbapenemase genes to endogenous soil bacteria through sludge disposal. Moreover, the removal efficiency for total bacteria (assessed by 16S rRNA genes) through chlorination disinfection was higher (*p* < 0.05) than bacteria harboring carbapenemase genes. The removal efficiency for 16S rRNA gene by chlorination was 90.5 ± 7.5% versus 52.4 ± 4.1% for *bla*_KPC-2_, 12.9 ± 2.8% for *bla*_GES-1_, and 4 ± 0.3% for *bla*_IMP-1_. This finding suggests that some bacteria harboring antibiotic resistance genes may be co-resistant to chlorination, as suggested by previous research [42,43]. However, resistance to chlorination is unlikely to be mechanistically related to antibiotic resistance. Disinfection is often thought to destroy most pathogens in WWTP effluents, and chlorination is the most common treatment used as compared to ultraviolet (UV) or ozonation disinfection. However, the results of this study showed that disinfection by chlorination could not achieve a significant reduction of carbapenemase genes in the WWTP, which is also consistent with previous research on *tet*-ARGs [44]. Although the abundance of carbapenemase genes were reduced somewhat through the WWTP, the continuous discharge from the WWTPs was still a potential route of carbapenemase genes into the environment.

### Flow of carbapenemase genes through various processing stages

To study the fate or removal of carbapenemase genes throughout different WWTP stages, the flow rates (gene copies/day) of carbapenemase genes and 16S rRNA genes (as a surrogate for total bacteria) were determined by multiplying the corresponding gene concentrations by volumetric flow rates. There was a significant replication of carbapenemase genes and 16S rRNA genes in the biological treatment units (Fig 1), where sewage biodegradation and microbial growth occurred. Consistent with the concentration of carbapenemase genes obtained by bacterial settling, a relatively high total discharge of carbapenemase genes ((1.3 ± 0.4) × 10^17^ to (2.3 ± 0.2) × 10^18^ copies/day) occurred in dewatered sludge. Thus, compared with influent values, a larger amount of carbapenemase genes in the effluent plus waste DS were discharged through the WWTP, as shown in Fig 1. Specifically, the total load (effluent plus waste DS) of the *bla*_KPC-2_ and *bla*_IMP-1_ genes discharged from the WWTP were 2.7 and 1.7 orders greater than influent values, respectively, and the *bla*_GES-1_ gene was 6.2-fold (0.2-fold in the FE and 6.0-fold in the DS) higher than the influent load. These results indicated an overall increment of carbapenemase genes within the WWTP.

### Bacterial isolates and antimicrobial susceptibilities

**Fig 2:**
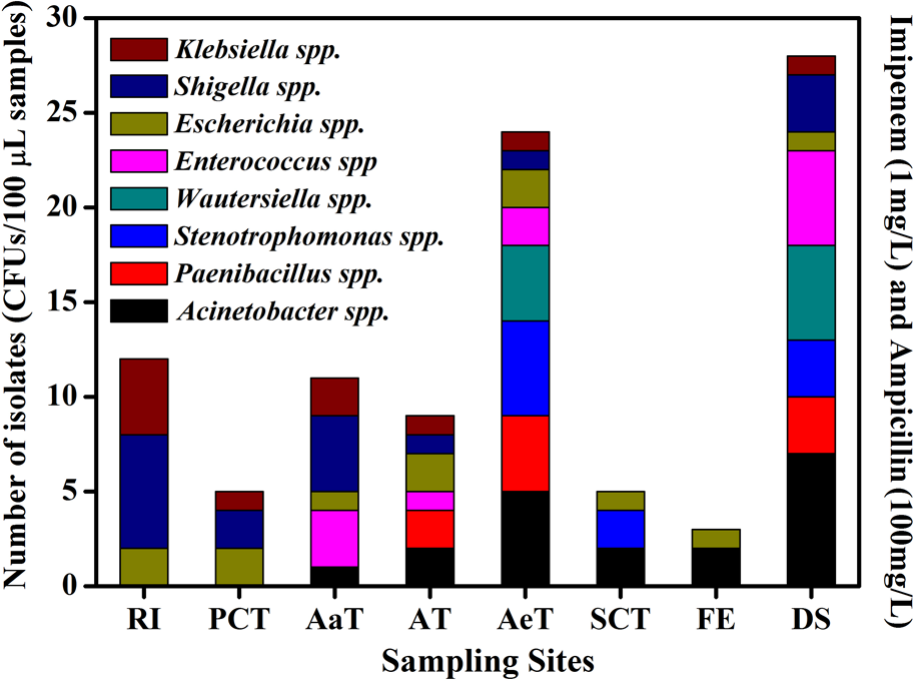
Distribution of KPC-2 producing strains at different sampling sites and with selective pressure applied. Please refer to Fig 1 for plant configuration and stage abbreviations. The numbers of isolates was represented as the CFUs per 100 μL samples from each sampling site. Species identification of these isolates was determined by 16S rRNA gene sequencing (% similarity): *Klebsiella spp*. (99.3%-99.6% identity to *K*. *oxytoca* strain JCM1665); *Enterococcus spp*. (97.4%-98.5% identity to *E*. *faecium* strain gp34); *Acinetobacter* spp. (98.2%-99.3% identity to *A*. *seohaensis* strain SW-100); *Escherichia spp*. (99.7%-99.9% identity to *E*. *coli* O157); *Shigella spp*. (99.4%-99.8% identity to *S*. *sonnei* strain CECT4887); *Stenotrophomonas spp*. (98.2%-98.8% identity to *S*. *maltophilia* R551-3); *Wautersiella spp*. (97.5%-98.1% identity to *Wautersiella* sp. MBG55); and *Paenibacillus spp*. (99.0%-99.2% identity to *Paenibacillus sp*. 1–9).

The emergence of KPC-2 has become an established health threat that represents a major challenge for the treatment of infectious diseases. To date, KPC-2 has been identified in isolates of *Klebsiella spp*. [15,45–47], *Pseudomonas spp*. [48–50], *Serratia spp*. [51], *Enterobacter spp*. [5,52,53], *Acinetobacter spp*. [54] and *Stenotrophomonas spp*. [55]. Here, a total of 97 KPC-2-producing bacterial strains were isolated from the WWTP, as shown in Fig 2. According to the results of the 16S rRNA gene sequencing, most of KPC-2 producing isolates were opportunistic pathogens, including 10.3% *Klebsiella spp*., 11.3% *Enterococcus spp*., 19.6% *Acinetobacter spp*., 12.4% *Escherichia spp*., 17.5% *Shigella spp*., 10.3% *Stenotrophomonas spp*., and 9.3% *Wautersiella spp*. Moreover, *bla*_KPC-2_ positive strains of *Paenibacillus spp*. were also isolated from the WWTP. It is worth mentioning that the *bla*_KPC-2_ gene was identified for the first time in *Enterococcus* spp. in this study. In order to make this finding more proved, all of the *Enterococcal* isolates were further tested for phenotypic characteristics by conventional methods [56–58]. Basic microbiological tests demonstrated that the strains were Gram-positive cocci, catalase-negative, good growth on bile esculin azide agar, and are able to grow in 6.5% (w/v) NaCl broth and at pH 9.6, which indicated that these KPC-2-producing isolates belong to the genus *Enterococcus*. Moreover, the full length sequence of 16S rRNA of these isolates were 97.4–98.5% identical to that of *Enterococcus faecium* strain (Gene ID: KM495939.1). Of all these *bla*_KPC-2_-positive isolates, a high proportion (74.2%) were isolated from the biological units, and the remaining 25 isolates (25.8%) were isolated from other units (including the WWTP effluent).

Notably, the *bla*_KPC-2_ genes were identified in indigenous bacteria of *Paenibacillus spp*., which are commonly found in the environment. To our knowledge, this is the first study to report that *bla*_KPC-2_ genes are being harbored by *Paenibacillus spp*. strains. PCR amplification followed by sequencing showed that the *bla*_KPC-2_ genes in the indigenous bacteria were completely consistent with that of the strains of the opportunistic pathogen *Klebsiella* isolated from the influent of the WWTP. These data suggested that the *bla*_KPC-2_ genes could be harbored by pathogens, opportunistic pathogens and even indigenous environmental bacteria. Furthermore, the fact that KPC-2-producing strains were isolated from the WWTP effluent is worrying, as this could contribute to the occurrence and spread of *bla*_KPC-2_ genes in the receiving environment. The environmental isolates harboring *bla*_KPC-2_ genes were highly resistant to β-lactams, including ampicillin (64—>256 mg/L), imipenem (8—>256 mg/L), meropenem (16—>256 mg/L) and ertapenem (8—>256 mg/L) (Table 3). All of the isolates were also found to be resistant to tetracycline, streptomycin, and gentamicin, and 75% of the isolates were resistant to ciprofloxacin. Obviously, these multidrug resistant bacteria, which occur in the WWTP, represent potentially serious risks for human health once they propagate through the environment [59].

**Table 3:** Resistance profiles of KPC-2-producing bacteria isolated from the WWTP for key antibiotics.

Antibiotic	MIC range (mg/L) for							
	*Klebsiella spp*. (n* = 10)	*Enterococcus spp*. (n = 11)	*Stenotrophomonas spp*. (n = 10)	*Acinetobacter spp*. (n = 19)	*Escherichia spp*. (n = 12)	*Shigella spp*. (n = 17)	*Wautersiella spp*. (n = 9)	*Paenibacillus spp*. (n = 9)
**Imipenem**	64 to >256	8 to 64	32 to 256	64 to >256	32 to >256	32 to 64	16 to 128	8 to 128
**Meropenem**	32 to 256	32 to 128	64 to >256	≥256	32 to 128	16 to >256	32 to 128	16 to 128
**Ertapenem**	64 to >256	16 to 128	64 to >256	64 to >256	8 to 64	8 to 128	64 to >256	8 to 128
**Ceftazidime**	128 to >256	64 to 128	≥256	128 to >256	64 to >256	128 to >256	64 to 128	64 to >256
**Ampicillin**	>256	128 to >256	≥256	≥256	≥256	128 to 256	128 to >256	64 to 128
**Streptomycin**	64 to >256	32 to 128	128 to >256	128 to >256	32 to >256	32 to 128	64 to >256	32 to >256
**Tetracycline**	4 to >256	8 to 16	8 to 64	32 to 256	8 to 128	4 to 64	8 to 64	4 to 16
**Gentamicin**	16 to >256	8 to 32	8 to 128	16 to 128	8 to 256	8 to 32	8 to >256	4 to 64
**Ciprofloxacin**	4 to >256	2 to 32	0.5–8	8 to 128	2 to 64	4 to 16	0.5 to 64	2 to 16

The minimum inhibitory concentrations (MICs) of the antibiotics were determined using the broth microdilution method with 14 gradient dilutions (from 0.03 mg/L to 256 mg/L). MICs were read as the lowest concentration of each antibiotic at complete (100%) growth inhibited compared to control cultures. Results are shown as the range of MIC values among all the isolates of each bacterial genera.

*, number of isolates.

### Conjugative transfer of the IncF plasmid harboring the *bla*_KPC-2_ gene

The *bla*_KPC-2_ gene was detected by PCR in the transconjugant *E*. *coli* J53 strain, which suggested that the investigated *bla*_KPC-2_ genes were located on a conjugative plasmid. The results of the sequencing and BLAST analyses showed that the *bla*_KPC-2_ genes in transconjugants were 100% identical (892 bp) to that from the donor strains. Moreover, the *E*.*coli* J53 transconjugants were resistant to the broad-spectrum carbapenems, as was the environmental donor isolate (Table 4), and simultaneously acquired resistance to ampicillin (>256 mg/L), streptomycin (128 mg/L), gentamicin (>256 mg/L) and ceftazidime (>256 mg/L). These results indicated that *bla*_KPC-2_ genes in transconjugants were derived from the KPC-2-producing donors.

**Table 4:** Resistance profiles of KPC-2 for *Klebsiella* isolates, *E*.*coli* J53 harboring *bla*_KPC-2_ gene, and the *E*.*coli* J53 recipient strain.

Antibiotics	MIC (mg/L) of.		
	*Klebsiella* donor strain	Transconjugant *E*.*coli* J53	*E*.*coli* J53 recipient strain
**Ampicillin**	>256	>256	4
**Kanamycin**	>256	128	0.06
**Tetracycline**	256	4	0.03
**Imipenem**	256	256	0.03
**Meropenem**	>256	>256	0.03
**Streptomycin**	>256	128	4
**Ciprofloxacin**	256	0.5	0.06
**Ceftazidime**	>256	>256	0.06
**Gentamicin**	>256	>256	0.12

The MICs of the antibiotics were determined using the broth microdilution method. And MICs were read as the lowest concentration of each antibiotic at complete (100%) growth inhibited compared to control cultures.

Furthermore, the *bla*_KPC-2_-positive plasmid belonged to the IncF incompatibility group that usually provides antimicrobial resistance determinants for the bacterial host [60]. Numerous studies have indicated that IncF plasmids are associated with the abrupt worldwide emergence of clinically relevant extended-spectrum β-lactamases (ESBLs), such as KPC, NDM-1 and CTX-M-15 [61–65]. More worryingly, IncF plasmids are frequently encountered in clinical *Enterobacterial* pathogenic strains, such as *E*. *coli* [66,67], *S*. *enterica* [68], *K*. *pneumonia* [69], *Y*. *pestis* and *Y*. *pseudotuberculosis* strains [70]. Additionally, it is noteworthy that the IncF-prototype plasmids are the fertility factor F of *E*. *coli* [71,72], and these plasmids are able to autonomously transfer among bacteria [73,74].

More interestingly, the transfer frequency (i.e., the numbers of transconjugants per recipient cell) of the *bla*_KPC-2_ IncF plasmid was 7.51 × 10^−5^, which was higher than the transfer frequency of RP4 plasmid (IncP group) from *E*. *coli* to *S*. *enterica* ((3.30 ± 0.52) × 10^−6^ transconjugants/recipient). Numerous studies have also revealed that IncF plasmids are self-transferable with a high transfer frequency (ranging from 10^−7^ to 10^−2^) [75–77]. These results suggest that more attention should be paid to the dissemination of IncF plasmids harboring ARGs, and specifically to *bla*_KPC-2_ high-risk genes. Multidrug-resistant strains harboring the *bla*_KPC-2_-positive self-transferable plasmid prevailed in the WWTP, highlighting a potential threat to public health. Therefore, further studies are needed to investigate the presence of these carbapenemase-producing strains in various environmental arenas.

## Conclusion

This study found that carbapenemase genes (the *bla*_KPC-2_, *bla*_GES-1_ and *bla*_IMP-1_ genes, but not *bla*_VIM-2_ and *bla*_OXA-48_ genes) and carbapenemase gene positive bacteria prevailed through each processing stage of a Chinese wastewater treatment plant, indicating that WWTPs may be a potential point source of carbapenem resistance to the environment. The substantial replication and discharge of carbapenemase genes, especially through dewatered sludge, emphasized that further treatment is needed to mitigate proliferation and propagation of bacteria in the sludge. Furthermore, the presence of high numbers of KPC-2-producing bacteria in the WWTP may have ecological and public health implications. Notably, the investigated *bla*_KPC-2_ genes were confirmed to disseminate resistance to indigenous bacteria, which increased the risk of *bla*_KPC-2_ gene propagation in the environment receiving effluent discharge. This finding emphasizes the need of further studies on the transfer of these high-risk resistance genes to humans through land fertilization and water usage.
